# Biocatalytic desulfurization of thiophenic compounds and crude oil by newly isolated bacteria

**DOI:** 10.3389/fmicb.2015.00112

**Published:** 2015-02-13

**Authors:** Magdy El-Said Mohamed, Zakariya H. Al-Yacoub, John V. Vedakumar

**Affiliations:** Biotechnology, Research and Development Center, Saudi Aramco, DhahranSaudi Arabia

**Keywords:** biodesulfurization, crude oil, thiophenic compounds, bacteria, 4S pathway

## Abstract

Microorganisms possess enormous highly specific metabolic activities, which enable them to utilize and transform nearly every known chemical class present in crude oil. In this context, one of the most studied biocatalytic processes is the biodesulfurization (BDS) of thiophenic sulfur-containing compounds such as benzothiophene (BT) and dibenzothiophene (DBT) in crude oils and refinery streams. Three newly isolated bacterial strains, which were affiliated as *Rhodococcus* sp. strain SA11, *Stenotrophomonas* sp. strain SA21, and *Rhodococcus* sp. strain SA31, were enriched from oil contaminated soil in the presence of DBT as the sole S source. GC-FID analysis of DBT-grown cultures showed consumption of DBT, transient formation of DBT sulfone (DBTO_2_) and accumulation of 2-hydroxybiphenyl (2-HBP). Molecular detection of the plasmid-borne *dsz* operon, which codes for the DBT desulfurization activity, revealed the presence of *dszA*, *dszB*, and *dszC* genes. These results point to the operation of the known 4S pathway in the BDS of DBT. The maximum consumption rate of DBT was 11 μmol/g dry cell weight (DCW)/h and the maximum formation rate of 2-HBP formation was 4 μmol/g DCW/h. Inhibition of both cell growth and DBT consumption by 2-HBP was observed for all isolates but SA11 isolate was the least affected. The isolated biocatalysts desulfurized other model DBT alkylated homologs. SA11 isolate was capable of desulfurizing BT as well. Resting cells of SA11 exhibited 10% reduction in total sulfur present in heavy crude oil and 18% reduction in total sulfur present in the hexane-soluble fraction of the heavy crude oil. The capabilities of the isolated bacteria to survive and desulfurize a wide range of S compounds present in crude oil are desirable traits for the development of a robust BDS biocatalyst to upgrade crude oils and refinery streams.

## INTRODUCTION

Microbes have been in contact with crude oil since early stages of formation and maturation in different oil reservoirs. During this long history of contact, microorganisms have evolved enormous metabolic activities, which enabled them to utilize and transform nearly every chemical class in crude oil for their survival.

Sulfur is the third most abundant element in crude oil and heterocyclic thiophenic compounds are substantially the most abundant form of organic sulfur encountered in crude oils (Van Hamme et al., 2003). Global trends and regulations to reduce sulfur in different fuel streams to reduce harmful SO_2_ emissions from transportation vehicles have become stringent and sulfur has become a key factor determining the price of crude. The demand for ultra-low S fuels is growing rapidly and exceeds current capacity of refineries. The conventional hydrodesulfurization (HDS), which runs under severe operating conditions, is the most common technology that has been implemented in refineries for S removal. Although HDS is capable of achieving lower S levels, this technology suffers from high capital and operating costs as well as low efficiency in desulfurizing some refractory S-species present in crude oils and refinery streams, such as 4,6-dimethyldibenzothiophene (4,6-DMDBT; Babich and Moulijn, 2003). This circumstance has prompted researchers worldwide to explore other routes for desulfurization of fossil fuels.

Biodesulfurization (BDS), a microbial-mediated process for specific S removal from thiophenic compounds encountered in crude oils such as benzothiophene (BT), dibenzothiophene (DBT), and their alkylated homologs, has been reported in numerous microbial species. Bacterial species harboring the BDS activity have been seen as potential biocatalysts for developing a non-conventional process for desulfurization of crude oils (Monticillo, 2000; Kilbane, 2006; Xu et al., 2009). The desulfurization spectrum of the reported bacterial species against thiophenic compounds varies; the majority can desulfurize either DBTs or BTs, while only a few species are capable of desulfurizing both BTs and DBTs (Tanaka et al., 2002; Alves et al., 2005; Kilbane, 2006; Aggarwal et al., 2013; Wang et al., 2013). The BDS of DBT follows a biocatalytic pathway known as “4S,” which involves four S oxidized chemical intermediates and requires four moles of NADH (Aggarwal et al., 2013).

The genetics and biochemistry of DBT desulfurization have been elucidated in a wide range of bacterial species and have been shown to be controlled by three plasmid born genes *dszABC* and one chromosomal gene *dszD* (Piddington et al., 1995; Gupta et al., 2005). These genes code for: DszC monooxygenase, which catalyzes two consecutive oxidation steps of DBT into DBT-sulfoxide (DBTO); and then to DBT-sulfone (DBTO_2_), DszA monooxygenase, which catalyzes the oxidative C-S bond cleavage in DBTO_2_ forming 2-(2^′^-hydroxyphenyl) benzene sulfinate (HPBS); DszD oxidoreductase, which delivers the reducing equivalent (FMNH_2_) required for the function of DszC and DszA; and DszB desulfinase, which catalyzes the conversion of HPBS into 2-hydroxybiphenyl (2-HBP) and sulfite (Oldfield et al., 1997). The released sulfite is further oxidized into sulfate by sulfite oxidoreductase (SOR; Aggarwal et al., 2012). The BDS of BT also follows pathways that involve formation of four S-containing intermediates and requires two moles of NADH. The enzymes and genes involved in the desulfurization of BT are different from those operating in the desulfurization of DBT (Aggarwal et al., 2013; Wang et al., 2013). In BDS, only C-S oxidative bond cleavage takes place to release the S atom as sulfate and the carbon skeleton of the thiophenic compound remains intact as a phenolic end product. Accordingly, in the BDS process the thiophenic compound serves only as the sole S source for microbial growth and the phenolic end product preserves the caloric value of the fuel (Monticillo, 2000; Kilbane, 2006; Aggarwal et al., 2013). It is known that both of the end products of DBT desulfurization, namely sulfate and 2-HBP, exert regulatory effects on the BDS, which in turn result in limited cell growth and low desulfurization activity. 2-HBP exerts feedback inhibition on the enzymes of the 4S pathway while sulfate is known to exert repression of the *dsz* promoter (Mohebali and Ball, 2008). In our recent study using *Rhodococcus erythropolis* IGTS8, it was demonstrated that the concentrations of 2-HBP that led to 50% reduction in enzyme activities (IC_50_) for DszA, DszB, and DszC are much less than for the cytoplasmic 2-HBP concentration. This data suggested that the inhibition of Dsz enzymes by 2-HBP is responsible for the observed reduction in biocatalyst activity concomitant with 2-HBP generation (Abin-Fuentes et al., 2013).

Advancements in strain improvement via gene manipulation and recent understandings in bioprocess development have resulted in enhanced BDS process for removal of sulfur from model compounds (Kilbane, 2006; Li et al., 2008, 2009; Pan et al., 2013; Abin-Fuentes et al., 2014). *In silico* analysis of a stoichiometric flux-based model for sulfur metabolism and reconstruction of genome-scale metabolic network, studies in *R. erythropolis* have proposed that increase in the activity of SOR and decrease in the activity of sulfite reductase (SR), coupled with the enzymes operating in the 4S pathway and cofactors, are critical in increasing the BDS rate significantly (Aggarwal et al., 2011a,b, 2012). These findings supported previous reports, which postulated that the overall BDS activity is dependent on both the genetics and level of expression of the *dsz* genes as well as on other host-related contributions, which are needed for functioning of the desulfurization pathway (Kilbane, 2006). Despite all these novel and successful approaches, the achieved BDS rate is still far by two orders of magnitude to develop a commercial BDS for crude oils and refinery streams (Monticillo, 2000; Kilbane, 2006; Xu et al., 2009; Lin et al., 2010).

## MATERIALS AND METHODS

### CHEMICALS AND BIOCHEMICAL

All chemicals and biochemicals were of the highest quality commercially available and were purchased from Sigma-Aldrich (Germany), Promega (USA), Qiagen (Germany), Thermo-Fisher Scientific (Germany), and Oxoid (England).

### CULTURE MEDIA

A chemically defined medium (CDM) containing the following S-free chemicals per liter was used throughout the experimental scheme unless other modification is specified: 5.6 *g* K_2_HPO_4_, 1.08 *g* KH_2_PO_4_, 0.5 *g* NH_4_Cl, 200 mg MgCl_2_.6H_2_O, 20 mg CaCl_2_^.^2H_2_O, 3 mg FeCl_2_.4H_2_O, 0.5 mg ZnCl_2_, 0.5 mg MnCl_2_, 0.2 mg CuCl_2_, 0.1 mg CoCl_2._ 6H_2_O, 0.1 mg NiCl_2_.6H_2_O, 0.1 mg H_3_BO_3_, 0.2 mg Na_2_MoO_4_.2H_2_O, 1 μg biotin, 0.6 μg *p*-aminobenzoic acid, 1 μg niacine, 2 μg pyridoxamine-HCl, 1 μg Ca-panthothenate, 1 μg cyanocobalamin, and 1 μg thiamine dichloride. DBT was added as the bioavailable S source at final concentration of 0.5 mM (from stock solution of 0.5 M DBT in ethanol) and glycerol (10 g) was added as the sole carbon source. The final pH was adjusted to around 7.2. In some experiments, DBT was replaced by other S sources (0.5 mM) such as BT, 1-methyl DBT (1-MDBT), 4-methyl-DBT (4-MDBT), 2,3-dimethylDBT (2,3-DMDBT), 4,6- dimethyl DBT (4,6-DMDBT), dimethylsulfoxide (DMSO), and MgSO_4_. CDM-DBT supplemented with 1.5% of the agar was used for single colony isolation on plates. Control agar plates lacking DBT were used to check for growth on S compounds contained in agar. LB media were used for microbial enrichment and molecular characterization of biocatalysts.

### SOIL SAMPLES

Dozens of oil-contaminated soil samples were collected randomly from different sites at Abu Ali Island (Eastern Province/Saudi Arabia). The soil samples collected from the top and the subsurface layers (30 cm depth) of each site were mixed and dealt with as one sample during the time course of microbial enrichment scheme.

### MICROBIAL ENRICHMENT AND DBT-GUIDED ISOLATION OF BACTERIA

Four hundred milliliter of LB medium/1 L flask were inoculated with 20 *g* of homogenized soil sample and incubated at 30^∘^C under 150 rpm shaking for 1 week. 20 ml of the enriched cultures were sub-cultured into 400 ml of CDM-DBT and incubated under the same conditions for 10 days. The DBT-guided sub-culturing process was repeated three times in CDM-DBT. Bacterial cultures recovered from the last transfer were collected by centrifugation (12,000 rpm, 4^∘^C, 10 min), suspended in 10 ml of CDM-DBT containing 20% (vol/vol) glycerol, dispensed in 200 μl aliquots in eppendorf tubes and then were kept as original enrichments at -70^∘^C. For single colony isolation, 10-fold dilutions of the original enrichments were prepared and 300 μl were spread on CDM-DBT agar and CDM agar plates. Morphologically distinct colonies (designated as a series of SA1, SA2, SA3, and so on) from CDM-DBT agar were selected and transferred into 10 ml of CDM-DBT broth. The process of single colony isolation and transfer into CDM-DBT broth was repeated three times and the resulting DBT-adapted bacterial isolates were designated as SA1, SA11, and SA111, respectively, for the first soil sample, and so on for other soil samples.

### GROWTH CHARACTERISTICS AND UTILIZATION OF DIFFERENT C- AND S-SOURCES

The growth characteristics of three bacterial isolates SA11, SA21, and SA31 in CDM containing different C-sources (10 mM) such as glycerol, glucose, 2-HBP, ethanol, succinate, lactate, and citrate and different S-sources (0.5 mM) such as 1-MDBT, 4-MDBT, 2,3-DMDBT, 4,6-DMDBT, BT, DMSO, and MgSO_4_ were tested in CDM using an Automated Microbiology Growth Analysis System (BioscreenC, Oy Growth Curves Ab Ltd, Finland). The temperature and pH tolerance of the three bacterial isolates were tested in CDM containing glycerol and DBT as the bioavailable C and S source, respectively. The fresh cell weight (g FCW/L) and dry cell weight (g DCW/L) were determined after filtration of cultures using pre-weighted 0.2 μm filters (FCW) followed by drying at 100^∘^C for 48 h (DCW) and corrected for filtration of cell-free medium using identical filters.

### DETECTION OF BIODESULFURIZATION END-PRODUCT BY GIBBS ASSAY

Detection of 2-HBP, which is the desulfurization product of DBT, was followed as previously described (Konishi et al., 1997). Throughout the time course of utilizing DBT as the sole S-source, 2 ml samples were withdrawn from DBT-cultures and centrifuged at 12,000 rpm for 5 min. 1 ml of the cell-free supernatant was transferred into a clean eppendorf tube and the pH was adjusted to pH 8.0 using 1 M NaHCO_3_ or by adding 3 μl of NaOH (4M). 300 μl of the pH-adjusted supernatant was transferred into a microtiter plate and 3 μl of Gibbs reagent (10 mg of 2,6 –Dichloroquinone-4-Chlorimide dissolved in 10 ml ethanol) were added. The development of blue color within 30 min was indicative for the presence of 2-HBP. Standard curve for quantification of 2-HPB in the range of 1 to 10 mg L^-1^ was developed by measuring the intensity of the corresponding blue color at 610 nm (Kayser et al., 1993).

### INHIBITION OF DBT DESULFURIZATION BY THE END PRODUCT 2-HBP

Six cultures (OD 600_nm_ of 0.1) in CDM lacking any S-source were prepared for each bacterial isolate. The experiment started by adding 0.5 mM DBT to all cultures. Four flasks of DBT cultures received variable concentrations of 2-HBP (0.1, 0.3, 0.5, and 1 mM 2-HBP) at the beginning of the experiment. The flask number 5 was spiked with 0.5 mM 2-HBP when the OD 600_nm_ reached two during the log growth phase, whereas the flask number 6 served as control (no exogenous 2-HBP). Samples were taken throughout the whole experiments for testing growth inhibition and BDS activity testing (consumption of DBT). The growth inhibition in 2-HBP-treated cultures was measured by comparing the percentage of cells recovered [as colony forming units (CFU)] on DBT-agar plates after 48 h with cultures receiving no exogenous 2-HBP. The effect of 2-HBP on cell growth and BDS activity was retrieved from three independent experiments.

### TOLERANCE AND VIABILITY OF BIOCATALYST IN CRUDE OILS

Viability testing using CFU method and Gibbs assay were used to test the tolerance of the SA11 cells to survive in media containing different ratios of crude oil and to evaluate their BDS activity. Flasks containing CDM and different volumetric ratios of heavy crude oil (10, 30, and 50% oil in total volume of 100 ml) were inoculated with 5 ml of DBT-adapted SA11 biocatalyst (10^10^ cells/ml) and incubated at 30^∘^C under shaking (200 rpm). Flasks containing 100 ppm DBT in CDM and lacking crude oil served as control. At different time intervals (0, 24, 48, 96, 168, and 240 h), two identical flasks were terminated to test the viability and the BDS activity for each biocatalyst. The content of each flask was centrifuged for 30 min at 10,000 rpm and the collected cell pellet was suspended in 50 ml CDM containing 100 ppm DBT. Tenfold dilution of the cells up to 10^18^ was prepared and 200 μl of the last three dilutions (10^16^, 10^17^, and 10^18^) were spread on LB agar plates and incubated at 28^∘^C for CFU counting after 24 and 48 h. Samples from the suspended cells were tested after 20 min, 1 h, and 2 h for DBT/2-HBP conversion using Gibbs reagent. This experiment was run twice in two independent duplicates.

### DESULFURIZATION OF CRUDE OIL BY SA11 RESTING CELLS

SA11 bacterial biocatalyst was grown in 20 l bioreactor with CDM containing DBT as the sole S source and glycerol as the sole C source. Re-feeding with DBT and glycerol was repeated until optical density of 25 (OD 600_nm_) was reached. The DBT-adapted cells in the exponential phase were harvested by centrifugation and washed twice in CDM lacking any S source. Several runs in the bioreactor were conducted under the same conditions to prepare high yield of cells. To avoid carryover of exogenous DBT and 2-HBP into the crude oil assays, the cell pellet was re-suspended in the same CDM lacking any S source (OD 600_nm_ of 30, equivalent to 0.01 g DCW/ml) and was incubated under 200 rpm shaking at 25^∘^C. At different time intervals, samples were withdrawn and tested for the buildup of 2-HBP by Gibbs reagent. Once Gibbs reagent gave no blue color, six 1L flasks, each containing 360 ml of the resting DBT-adapted cells, were prepared. To each of the flasks, 40 ml of heavy crude oil (3% S) were added. The flasks were incubated under 250 rpm shaking at 30^∘^C and at different time intervals (2, 4, and 7 days) two flasks were terminated. Crude oil contents were collected after centrifugation of samples (40 min, 8500 rpm) and were analyzed for total S and S speciation.

### GC ANALYSIS FOR IDENTIFICATION OF BDS CHEMICAL INTERMEDIATES

The chemical intermediates of DBT transformation pathways were traced using GC-analysis (HP-6890 GC-FID). Cell-free supernatant was acidified to pH 2 and extracted with methylene chloride. The solvent was evaporated and the residue was dissolved in hexane. In other experiments, hexane extraction of DBT and metabolites thereof present in cells as well as in culture supernatant was performed for GC analysis. The GC was fixed with Trace TR-5 column (60 m, 0.25 mm ID, 0.25 mm film thickness, Thermo Scientific) and helium was used as carrier gas at 1.6 ml/min. The GC temperature program used an initial temperature of 80^∘^C for 4 min, raised to 250^∘^C at 8^∘^C/min, and held for 15 min. The temperature of the injector and the FID detector were set at 280 and 300^∘^C, respectively. A mixture consisting of DBT, DBT-sulfone, and 2-HBP (100 ppm, each) in hexane was prepared as a standard solution.

### TOTAL S AND S SPECIATION ANALYSIS

Total S in different crude oil samples was determined by X-ray fluorescence spectrometry (XRF) and S speciation in the hexane-soluble crude fraction was analyzed using two-dimensional gas chromatography (2D-GC) hyphenated with a sulfur selective detector (SCD).

### DNA EXTRACTION

Isolation and purification of genomic DNA was followed using Wizard Genomic DNA purification kit instruction (A1120, Promega). The extracted DNA was checked on 0.8% agarose-ethidium bromide gels and visualized using DNA imaging system (Bio-Rad). The quantity and quality of DNA was determined using Thermo Scientific NanoDrop^TM^ 2000c Spectrophotometer.

### MOLECULAR CHARACTERIZATION OF GENES ENCODING DBT/2-HBP CONVERSION

The detection of *dszABC* genes coding for the bioconversion of DBT into 2-HBP in the three isolates was followed using a set of primers described by Kayser et al. (2002). The following hot start thermal cycling was used in Tetrad 2 PCR thermal cycler (Bio-Rad): 96^∘^C, 5 min; 30 cycles of 96^∘^C for 30 sec denaturation, 50^∘^C, for 1 min primer annealing, and 72^∘^C for 3 min extension followed by one cycle at 72^∘^C for 10 min to complete extension. The amplified PCR products were separated using agarose gel electrophoresis (1% agarose with ethidium bromide).

### MOLECULAR IDENTIFICATION OF BIOCATALYSTS

The molecular scheme followed for identification and phylogenetic affiliation of biocatalysts SA11, SA21, and SA31 was based on amplification of eubacterial, nearly full-length, 16S rRNA gene using the sequence of the forward primer (16Sf) 5′-AGAGTTTGATCCTGGCTCAG-3^′^ and of the reverse primer (16Sr) 5^′^-AAGGAGGTGATCCAGCC-3^′^ as described by Weisburg et al. (1991). The obtained PCR products were confirmed by agarose gel electrophoresis. The purified 16S rRNA fragments were cloned in Promega pGEM-T Easy Vector System and Invitrogen DH5α chemically competent cells. 12 clones for each biocatalyst were picked for 16S rRNA sequencing. The sequences were submitted to Blast search for determination of the closet relative sequences in the GenBank database. At least 10 good sequences from each sample were used to sequence identity. The neighbor-joining phylogenetic trees were constructed using ClustalX program^1^ or^2^. DNA cloning and sequencing was done at Gas Technology Institute (GTI), Des Plaines, IL, USA^3^.

### DNA ACCESSION NUMBER

All DNA sequences reported in this study are deposited in a database under the following accession numbers: KM513655, KM513656, and KM513657.

## RESULTS

### ENRICHMENT AND ISOLATION OF BACTERIAL ISOLATES UTILIZING DBT AS S-SOURCE

The designed experimental scheme was planned to enrich, on complete LB medium, the majority of viable bacterial communities inhabiting oil-contaminated soil samples in the first screening run. This was followed by stressed screening in the presence of DBT as the sole S source in CDM. Tens of morphologically distinct bacterial colonies were selected from CDM-DBT agar plates and subjected to further purification steps. After successive transfers in CDM-DBT and Gibbs assay-guided purification protocol, three distinctive fast growing bacterial isolates (designated as SA11, SA21, and SA31) were selected for further investigation. The culture-free supernatants of the selected lead isolates gave a permanent blue color with Gibbs reagent and the intensity of the blue color increased in parallel with increased bacterial growth and elapse of time. SA11 and SA31 exhibited gram positive phenotype, whereas SA21 showed gram negative phenotype.

### GROWTH CHARACTERISTICS AND UTILIZATION OF OTHER C AND S SOURCES

**FIGURE 1:**
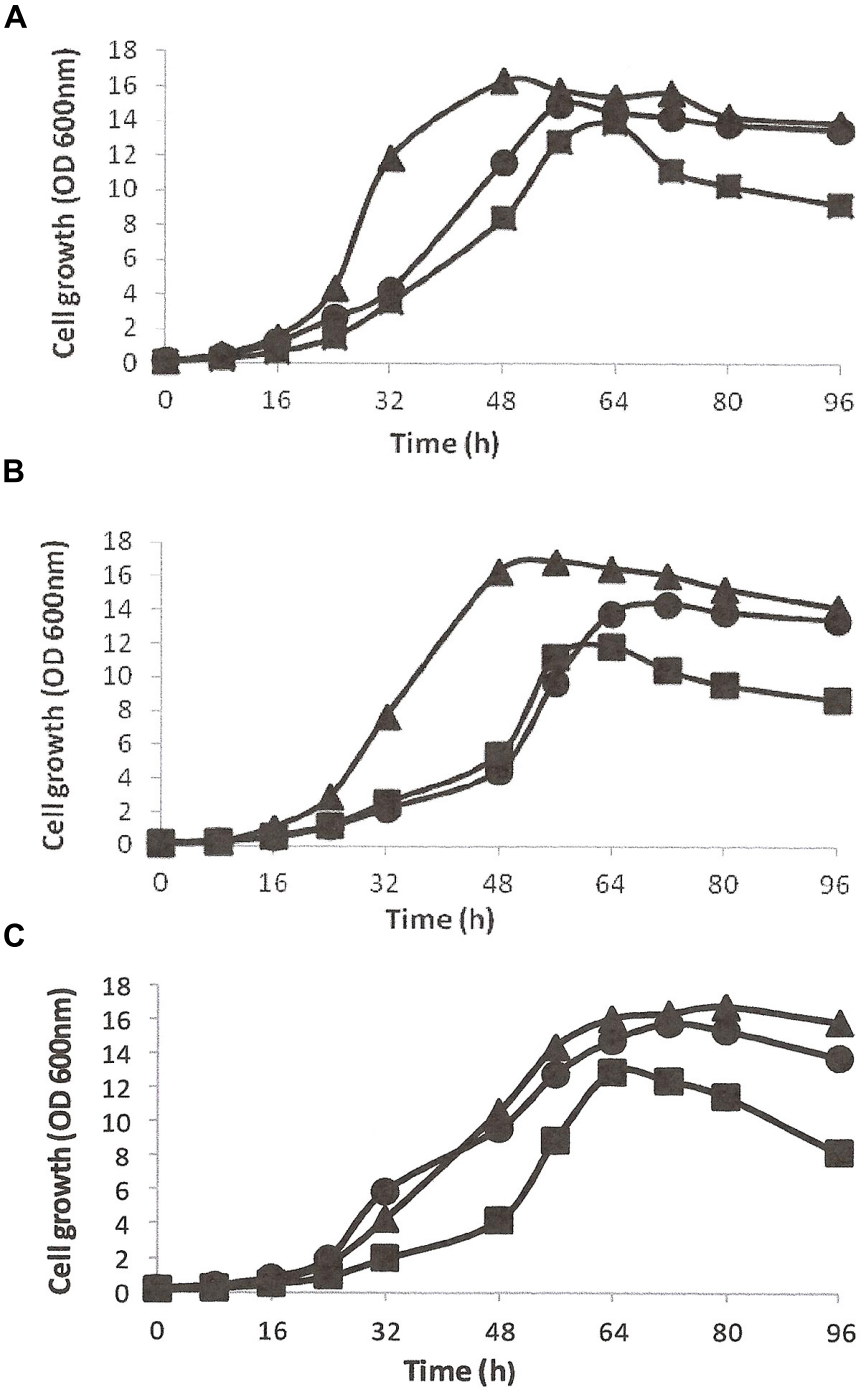
**Growth curves of bacterial isolates SA11 **(A)**, SA21 **(B)**, and SA31 **(C)** in CDM containing glycerol as the sole C-source and different S-sources (DBT▪, DMSO▴, and SO_4_•)**.

The growth and utilization of DBT (0.5 mM, ≈92 ppm) as the sole S-source in the presence of glycerol as the energy source was studied in CDM for all isolates (**Figures 1A–C**). DBT-adapted SA11 cells grew faster and exhibited higher growth rate than cells of SA21 and SA31 [values for growth rate constant (*k)* of 0.112 h^-1^, 0.092 h^-1^, and 0.096 h^-1^ corresponding to doubling time (*g)* of 6.2, 7.5, and 7.2 h, respectively]. When DMSO served as the bioavailable S source, all DMSO-adapted bacterial isolates exhibited higher growth rates and shorter doubling times (*k* values of 0.16, 0.14, and 0.13 h^-1^ for *g* values of 4.4, 4.9, and 5.2 h; respectively for SA11, SA21, and SA31). The growth of all isolates with MgSO_4_, as the sole S-source, was less than with DMSO, but still it was higher than with DBT (**Figure 1**). The cell yield with DBT was less than with both DMSO and SO_4_ for all isolates. It was observed that all bacterial isolates growing with DBT displayed very short stationary growth phase followed by fast decline phase. Other DBT alkylated homologs such as1-MDBT, 4-MDBT, 2,3-DMDBT, and 4,6-DMDBT served as good S-sources supporting cell growth of all biocatalysts. Neither SA 21 nor SA 31 strains showed any signs of growth when BT served either as the sole S-source in presence of glycerol or as the sole S- and C-source in absence of glycerol. In contrast, SA11 grew well with BT as S-source in presence of glycerol and no growth was observed when glycerol was omitted. However, DBT was better than BT as S-source in supporting growth of SA11 cells in presence of glycerol (OD_600__nm_ of around 12 and 8, respectively which resulted in cell yield of 3.9 and 2.5 g/L DCW, respectively). Glycerol was the preferred C-source for all isolates and resulted in high cell yield. All other tested C-sources except for 2-HBP were utilized, but the cell yield was less than that obtained with glycerol [for SA11, the cell yield (*g* DCW/L) of 3.9, 2.7, 3.4, 1.3, 3.0, and 0.2 was observed for glycerol, glucose, ethanol, succinate, lactate and citrate, respectively]. No signs for growth with 2-HBP were observed for all isolates even after 2 weeks of incubation. In CDM containing glycerol as the sole C-source and DBT as the sole S-source, DCW determinations were 16, 18, and 15% of the fresh cell weight (FCW) for SA11, SA21, and SA31, respectively. An average of 2–2.2 *g* FCW/L was equivalent to one unit of OD 600_nm_ for all bacterial isolates under the standard growth conditions with DBT and glycerol (at 30^∘^C and pH 7.2). All isolates showed optimum growth at 28–35^∘^C and survived at pH values 5.5–8.5, and grew substantially better at pH 7–7.5.

### DESULFURIZATION OF DBT AND IDENTIFICATION OF METABOLIC INTERMEDIATES

**FIGURE 2:**
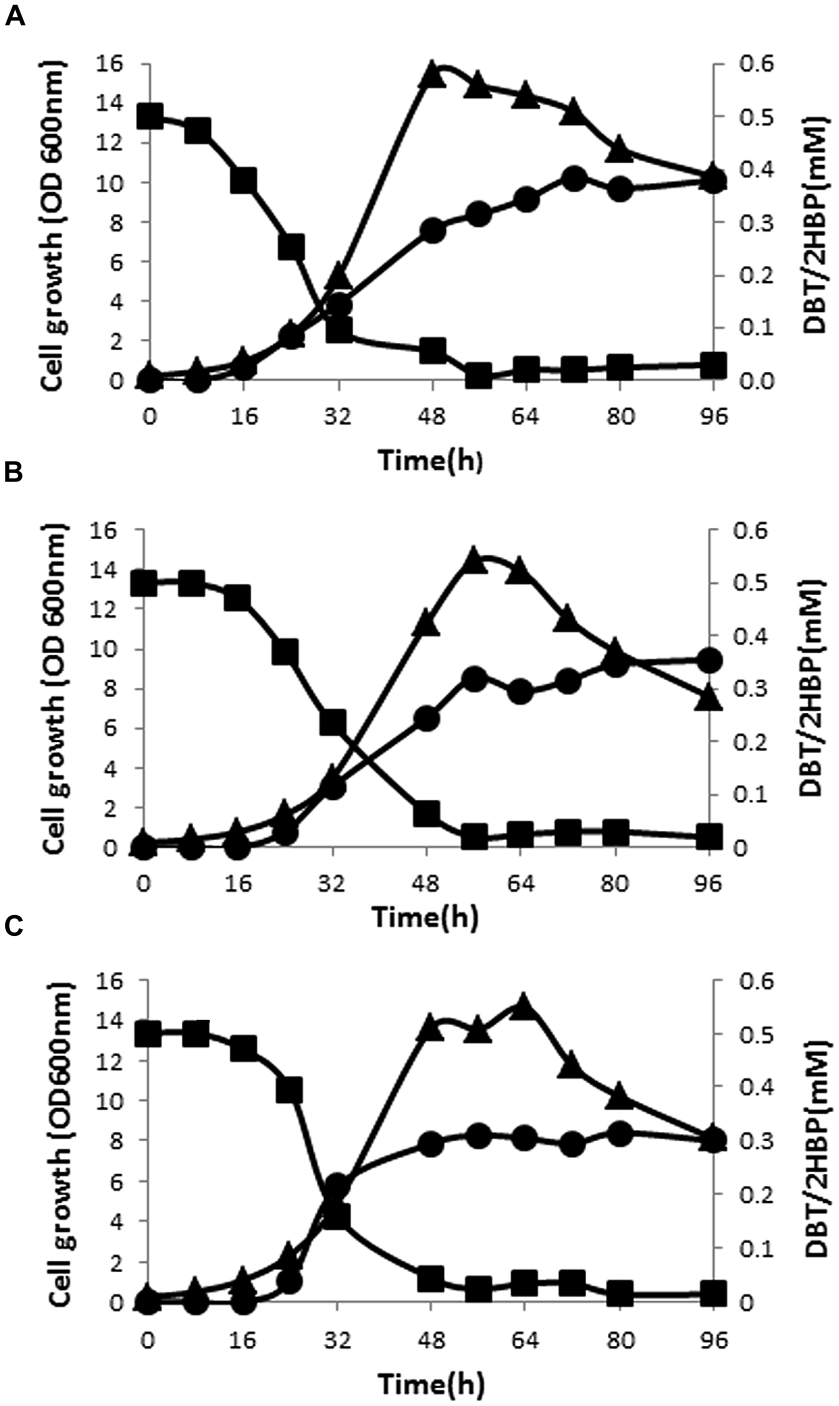
**Growth (▴), time course of DBT consumption (▪), and 2-HBP formation (•) by bacterial isolates SA11 (A), SA21 (B), and SA31 (C)**.

Preliminary result of testing cell-free supernatants of SA11 cultures with Gibbs reagent revealed formation of phenolic compound(s) when DBT or BT served as the sole S-source (formation of permanent blue color). GC-FID system was established to detect the intermediates of DBT desulfurization based on authentic samples. Extraction of DBT intermediates from cell-free culture supernatants as well as from the cells and analysis by GC-FID revealed the presence of intermediates matching DBT-sulfone (DBT-O2) and 2-HBP in cultures of all isolates. DBT-O2 was transiently detected in early samples while 2-HBP was persistent in all later samples. DBT sulfoxide (DBTO) and 2-(2^′^-hydroxyphenyl) benzene sulfinate (HBPS), proven intermediates in DBT-desulfurization pathway, were not traced in the analyzed samples due to the lack of standard samples. The time course of DBT consumption did not match 2-HBP formation for all bacterial biocatalysts (**Figures 2A–C**). At any time interval, the amount of detected 2-HBP was much less than the amount of the consumed DBT. The consumption of DBT started early while 2-HBP was detected later after 8–16 h. Although DBT was almost totally consumed, the produced and accumulated amount of 2-HBP was around 60–75% of the transformed DBT. The maximum consumption rate of DBT (11 μmol/*g* DCW/h) was observed at the early exponential growth phase and the maximum 2-HBP formation rate (4 μmol/*g* DCW/h) was reached in the mid-exponential growth phase for SA11. Although SA21 and SA31 exhibited nearly the same profile of DBT consumption and 2-HBP production, the observed rates were less by around 20% compared with SA11.

### EFFECT OF 2-HBP ON CELL GROWTH AND BDS ACTIVITY

The number of CFU recovered (on DBT-agar plates after 48 h) from 2-HBP-treated cultures was taken as a measure for the effect of 2-HBP on cell growth. Obviously, cultures of SA11, SA21, and SA31 bacterial isolates showed signs for growth inhibition at any tested exogenous concentration of 2-HBP when compared with control culture receiving no 2-HBP (**Table 1**). All cells recovered from cultures treated 0.5 or 1.0 mM 2-HBP failed to express BDS activity for simultaneous conversion of DBT into 2-HBP (no formation of blue color with Gibbs reagent) compared with untreated cultures. It was observed that the inhibition of the growth by 2-HBP is concentration dependent. All biocatalysts exhibited severe growth inhibition at 0.5 and 1 mM 2-HBP (accounting for around 65 and 100%, respectively). Less growth inhibition was observed at lower concentrations of 2-HBP. Remarkably, SA11 biocatalyst was the least inhibited biocatalyst, which exhibited around 30% growth retardation at 0.3 mM 2-HBP. DBT cultures of all isolates spiked with 0.5 mM 2-HBP during the log growth phase (at OD_600__nm_ of around 2–3) responded by sharp drop (of about 1.2–1.7 OD_600nm_ units) in growth within 1 h after addition.

**Table 1:** Inhibitory effect of 2-HBP on cell growth of DBT-desulfurizing bacterial isolates.

2-HBP (mM)	% of CFU recovered from 2-HBP-treated cultures		
	SA11	SA21	SA31
0.0 (control) 0.1 0.3 0.5 1.0	100 92 71 39 0.0	100 81 54 32 0.0	100 77 49 35 0.0

*The percentage of CFU recovered on plates (after 48 h) from 2-HBP-treated oxcultures (0.1–1.0 mM) was compared with cultures receiving no exogenous 2-HBP to express the extent of growth inhibition.*

### TOLERANCE, VIABILITY AND BDS ACTIVITY OF SA11 BIOCATALYST IN CRUDE OILS

**FIGURE 3:**
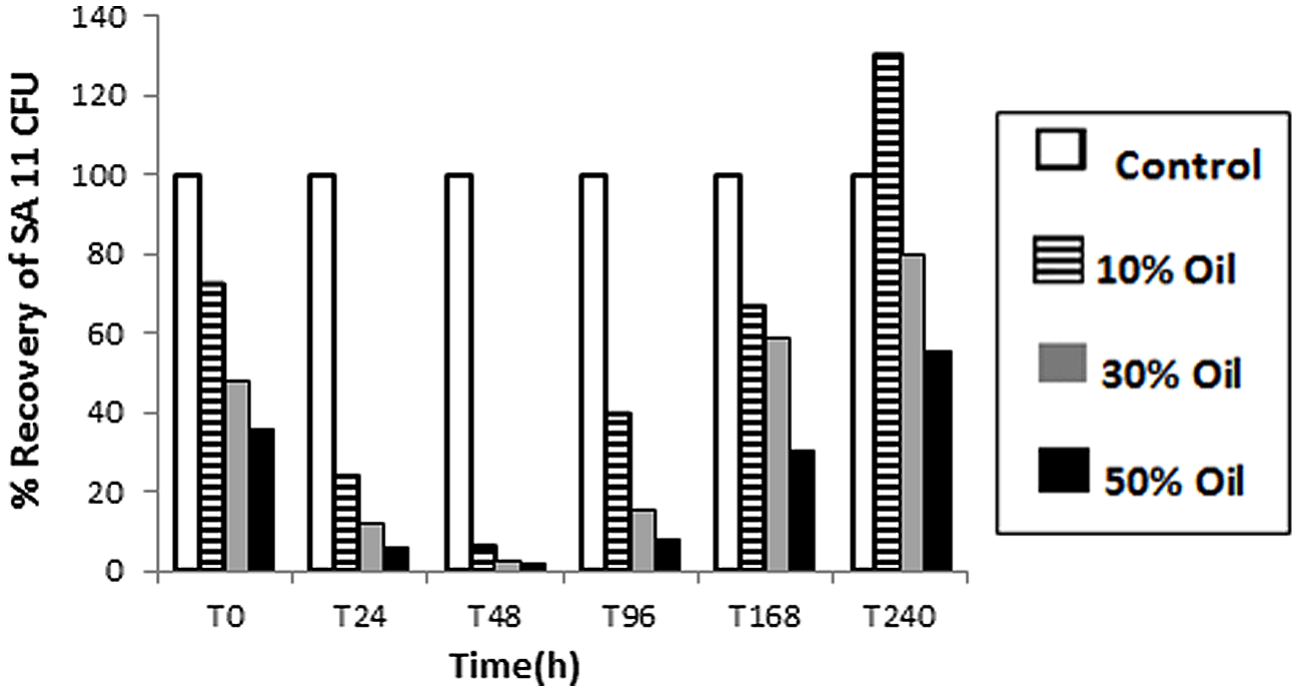
**Viability of SA11 cells in crude oil environment compared to control cultures containing DBT.** Viability is expressed as a % of cells recovered (CFU after 48 h on DBT-agar plates) from assays containing different crude oil concentrations (10–50% vol/vol) at different time intervals.

The tolerance and viability, as well as BDS activity, of SA11 biocatalyst were tested in CDM containing different concentrations of heavy crude oil. Obviously, the number (CFU detected on DBT-agar plates after 48 h) of recovered cells by centrifugation from oil containing cultures was much less than control cultures containing DBT (**Figure 3**). The decrease in the number of recovered cells runs in parallel with the increase in oil concentration from 10 to 50% and with the elapse of time until 48 h. Only less than 10% of the inoculated cells were recovered from crude oil containing cultures after 48 h. Thereafter, a remarkable gradual increase in the recovered cells was observed with elapse of time. The numbers of recovered cells after 240 h incubation were much higher than the corresponding numbers of cells recovered at 0 h from the same assay. The recovered colonies on DBT-agar plates exhibited similar phenotypic characteristics with the original SA11 isolate. Formation of oil/water tight emulsion was observed progressing with time and was more pronounced in assays containing more oil. In general, the portion of recovered cells from assays containing 10% oil was much higher than that from assays containing 50%. DBT culture supernatants of cells recovered from all crude oil treatments and tested with Gibbs reagent gave permanent blue color. This result indicates that the recovered cells retained the catalytic activity of converting DBT into 2-HBP (BDS activity).

### DESULFURIZATION OF HEAVY CRUDE OIL BY DBT-ADAPTED RESTING CELLS

The capability of DBT-adapted resting cells of the SA11 biocatalyst to desulfurize S-containing compounds present in heavy crude oil was tested at 10% oil in CDM lacking exogenous S-sources. The total S content in SA11- treated heavy crude oil was reduced by 10% after 1 week of incubation. Total S and S-speciation analyses of the hexane soluble fraction of the heavy crude oil revealed 18% total S reduction including a wide range of thiophenic compounds such as DBT, BT, and their alkylated derivatives (data not shown).

### MOLECULAR DETECTION OF THE *dsz* GENES CODING FOR DBT DESULFURIZATION PHENOTYPE

**FIGURE 4:**
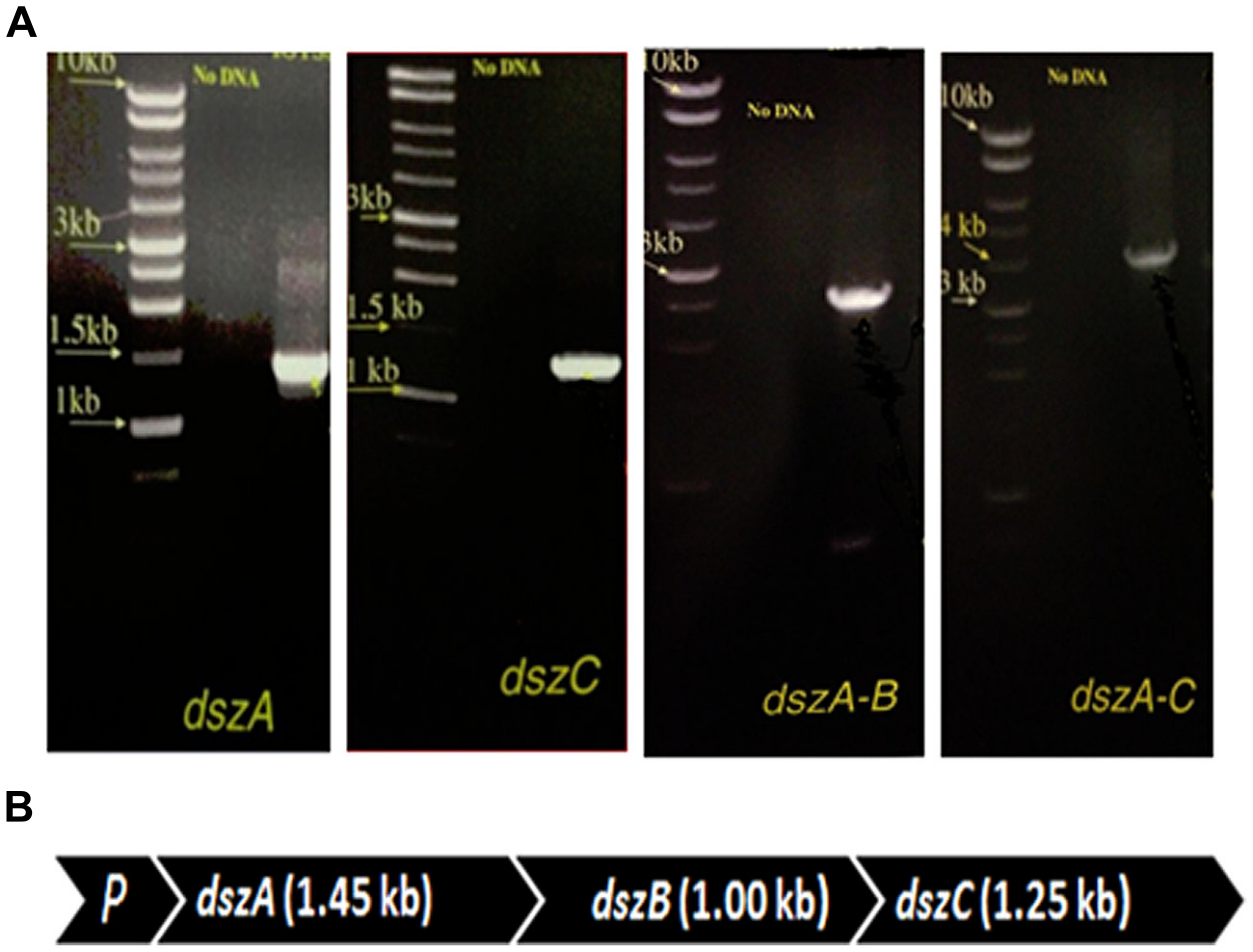
**(A)** Imaging of DNA fragments obtained from PCR assays of SA11 designed to amplify *dszA* (1.45 kb), *dszC* (1.25 kb), *dszAB* (2.5 kb), and *dszAC* (3.7 kb) genes, which code for the desulfurization of DBT via the 4 S pathway, **(B)** Organizational order of the *dszABC* genes in one operon in the newly isolated SA11. P, promoter.

The presence of *dszA, dszB*, *and dszC* genes encoding the BDS activity and their organization in the *dsz* operon were tested using combinations of primers in different PCR assays. The size of the obtained PCR products of *dszA* (1.45 kb), *dszC* (1.25 kb), *dszAB* (2.5 kb), and *dszAC* (3.7 kb) confirmed the presence of *dszABC* operon in SA11 and SA31 isolates (**Figures 4A,B**) with the same size and arrangement of genes in accordance with the known gene organization in DBT desulfurizing bacteria. PCR fragments of 1.45, 1.25, and 2.5 kb were detected with SA21 indicating the presence of *dsz* genes. However, some unspecific DNA bands were also amplified and the amplification of the 3.7 kb fragment was not successful.

### MOLECULAR IDENTIFICATION AND PHYLOGENETIC AFFILIATION OF BIOCATALYSTS

**FIGURE 5:**
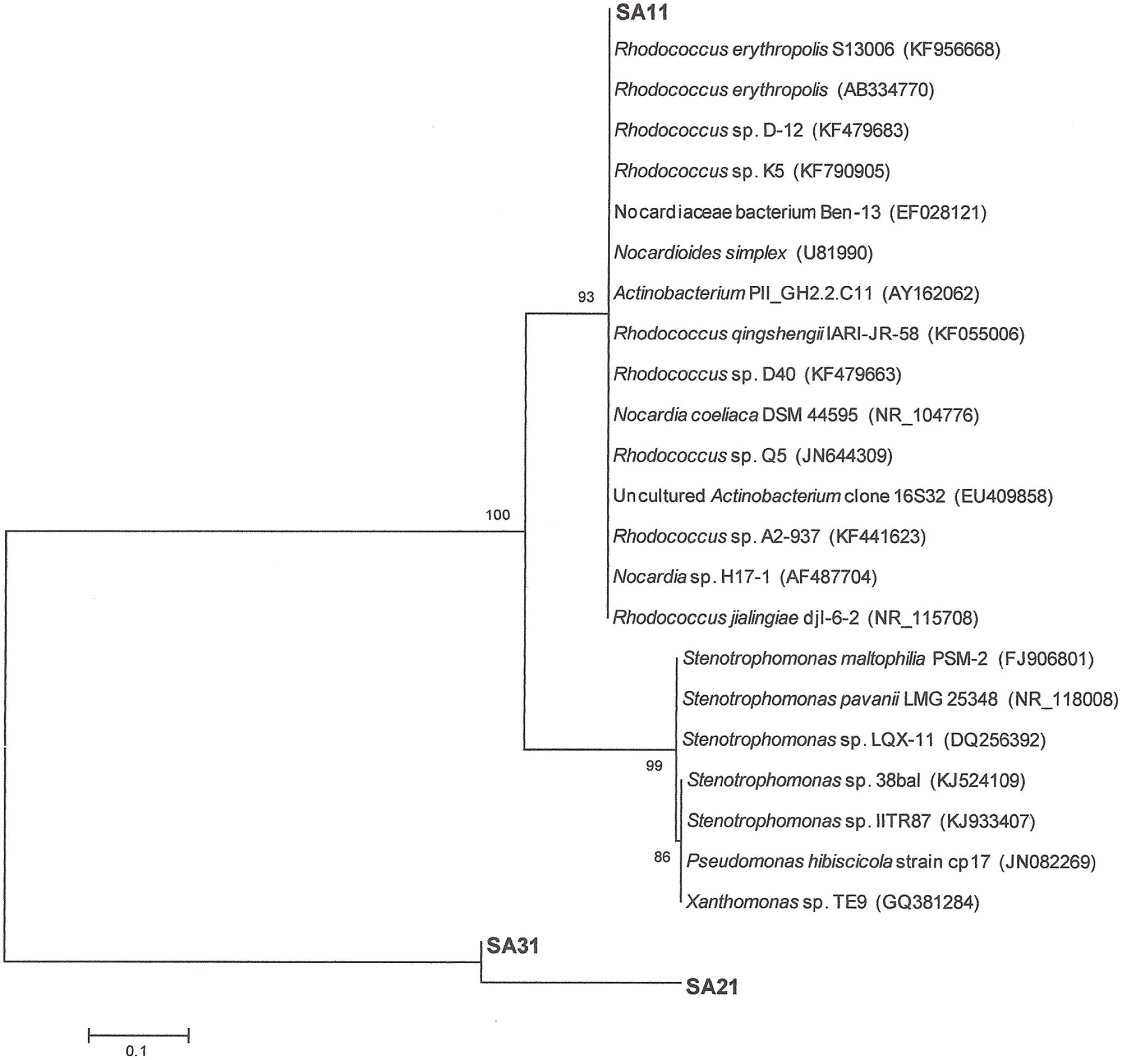
**Neighbor-joining consensus tree based on 16S rRNA gene sequence analysis showing the relationship between the strains SA11 and SA 31 and SA21 to the closest relatives in GenBank data base.** The bar represents 0.1 substitutions per site and bootstrap values (indicated at the nodes) were calculated from 1000 trees.

16S-rRNA gene amplification and sequencing was conducted for identification and phylogenetic alignment of the isolated SA11, SA21, and SA31 biocatalysts. Blast search sequence analysis of around 1000 bp of the amplified 16S rRNA from both the 5^′^ and 3^′^ termini in at least 10 clones for each biocatalyst was performed to align the biocatalysts to the most closely relatives in GenBank database. 16S rRNA sequences of SA11 and SA31 clones exhibited 99–100% sequence identity with different *Rhodococcus* spp., whereas 16S rRNA sequences from SA21 clones mainly exhibited 99% sequence identity with different *Stenotrophomonas* spp. The obtained data was used to construct phylogenetic trees indicating the affiliation to the closest relatives in GenBank in database (**Figure 5**).

## DISCUSSION

There is an increasing interest in developing biotechnological processes for various applications in oil and gas industry. Ample convincing data, highlighting the use of microbial cells or their metabolic products in exploration and production, as well as in improving and upgrading the quality of crude oil or refinery streams, have been reported. One of the most addressed biotechnological topics for improving fuel and oil quality is the microbial removal of sulfur in refinery streams and crude oil via a process known as BDS. Although evidence has been reported encouraging the development of BDS for treating refinery streams, little is known about its feasibility as a pre-refinery technology for treatment of crude oils. Moreover, progress in developing robust BDS biocatalysts, as well as in developing BDS bioprocess for crude oils, still is facing big hurdles such as low catalytic BDS rate, inhibition of biocatalyst by the end product 2-HBP, tight emulsion formation, separation of desulfurized crude, recovery of biocatalyst, and developing continuous system for high throughput bioprocesses. Characteristics of biocatalysts such as functionality and longevity in crude oil environment, broad desulfurization spectrum, less inhibition by the end products of BDS, and maintaining high and stable BDS rates, are crucial for the success of such bioprocesses. Accordingly, the present investigation aimed to isolate new biodesulfurizing bacterial biocatalysts capable of desulfurizing a wide range of thiophenic S compounds encountered in crude oil as well as having better characteristics in term of tolerance to high oil concentrations and less inhibition by 2-HPB, the end product of BDS.

### ENRICHMENT OF DBT DESULFURIZING BACTERIA FROM OIL-CONTAMINATED SOILS

The oil in the collected soil samples represents the residual weathered oil fraction remaining after both the natural physical and biological treatments prevailing in Abu Ali Island. The nature of such residual oil usually comprises recalcitrant heavy oil fractions, which are rich in asphaltenes, condensed polyaromatics, and heterocyclic compounds with elevated S contents (Huesemann, 1995; Mohamed et al., 2006). These compounds could serve as the bioavailable source for C, N, and S needed for the growth of indigenous microbial flora inhabiting oil contaminated environments. Accordingly, natural selection of microbial clones with high tolerance to oil components from such areas would help in fishing robust catalysts for BDS process. The employed DBT-guided enrichment scheme delivered diverse microbial isolates, which can metabolize DBT via different metabolic pathways. Most of the enriched bacterial population on agar plates, which gave no blue color or transient blue color with Gibbs reagent, possibly utilized DBT either as C or as C and S source via C–C and C–S bond cleavage following the known Kodama and/or angular dioxygenase pathways (Kropp and Fedorak, 1998; Nojiri et al., 2001). In contrast, the three bacterial isolates SA11, SA21, and SA31, which gave permanent blue color with Gibbs reagent, utilized DBT only as S source via the well-known 4S pathway (or BDS). This pathway, which was first reported in *R. rhodochrous* IGTS8 (Kilbane and Jackowski, 1992; Gallagher et al., 1993) and later in various bacterial genera (Kilbane, 2006), involves only C-S bond cleavage resulting in desulfurization of DBT and accumulation of the end product 2-HBP which gives blue color with Gibbs reagent.

### GROWTH CHARACTERISTICS AND DESULFURIZATION CAPABILITY OF BACTERIAL BIOCATALYSTS

The isolated DBT desulfurizing bacterial isolates showed maximum growth in the mesophilic temperature range and at neutral pH value. SA11 was the lead isolate in term of growth rates in presence of glycerol as C-source and different S-sources. Apparently, DMSO and SO_4_ are preferably utilized than DBT as S-source by all isolates. This might be due to the better solubility and bioavailability of these inorganic S-sources compared with DBT and/or due to the production of toxic DBT-intermediates, such as 2-HBP, which might limit/inhibit further growth during the time course DBT desulfurization. The observed fast drop in optical density after reaching the maximum growth with DBT would support the last explanation. Interestingly, although all isolates showed similar capabilities to utilize DBT and its alkylated homologs as S-source, their utilization of BT as S-source was dissimilar. Only isolate SA11 was capable of utilizing BT as S-source. Various microbial desulfurization patterns against DBTs and BTs have been reported. The majority of bacteria, which are capable of desulfurizing the symmetric heterocyclic S in DBTs such as many *Rhodococcus* spp. (strain IGTS8, Gallagher et al., 1993; strain D-1, Izumi et al., 1994; and strain H-2, Ohshiro et al., 1996) as well as *Agrobacterium* sp. MC501 (Constantí et al., 1996), *Nocardia* strain CYKS2 (Chang et al., 1998), *Mycobacterium pheli* WU-F1 (Furuya et al., 2001), *Bacillus subtilis* WU-S2B (Kirimura et al., 2001), and *Stenotrophomonas maltophilia* KHO1 (Ardakani et al., 2010) seem to be incapable of desulfurizing the asymmetric heterocyclic S in BT. The SA21 and SA31 bacterial isolates reported in this study, which presumably are affiliated to *Stenotrphomonas* and *Rhodococcus*, respectively, exhibited this desulfurization pattern. Similarly, BT desulfurizing bacteria such as *Gordonia* strain 213 (Gilbert et al., 1998), *Rhodococcus* sp. strain TO9 (Matsui et al., 2000), *Sinorhizobium* sp. strain KT55 (Tanaka et al., 2001), *Rhodococcus* sp. strain WU-K2R (Kirimura et al., 2002) and *Gordonia terrae* strain C-6 (Wang et al., 2013) seem to be lacking the enzymatic capability expressing DBT desulfurization phenotype. On the other hand, few bacterial strains such as *Paenibacillus* sp. strain A11-2 (Konishi et al., 2000), *Rhodococcus* sp. KT462 (Tanaka et al., 2002), *Mycobacterium goodi* X7B (Li et al., 2005), *Gordonia alkanivorans* strain 1B (Alves et al., 2005), and *Sphingomonas subacteria* T7b (Gunam et al., 2006), which exhibit broad desulfurization capability against DBTs and BTs have been reported. In almost all cases, the desulfurization of DBTs and BTs in this group is mediated by different enzymes. The SA11 isolate reported in this study, which presumably is a *Rhodococcus* sp., belongs to the BT and DBT desulfurizing bacteria. Bacteria from this group are valuable biocatalysts for developing bioprocess to desulfurize a wide range of thiophenic compounds, which constitute around 70% of the total organic S-in some crude oils (Monticillo and Finnerty, 1985).

### DBT DESULFURIZATION BY THE NEWLY ISOLATED BACTERIA OPERATES VIA THE 4S PATHWAY

**FIGURE 6:**
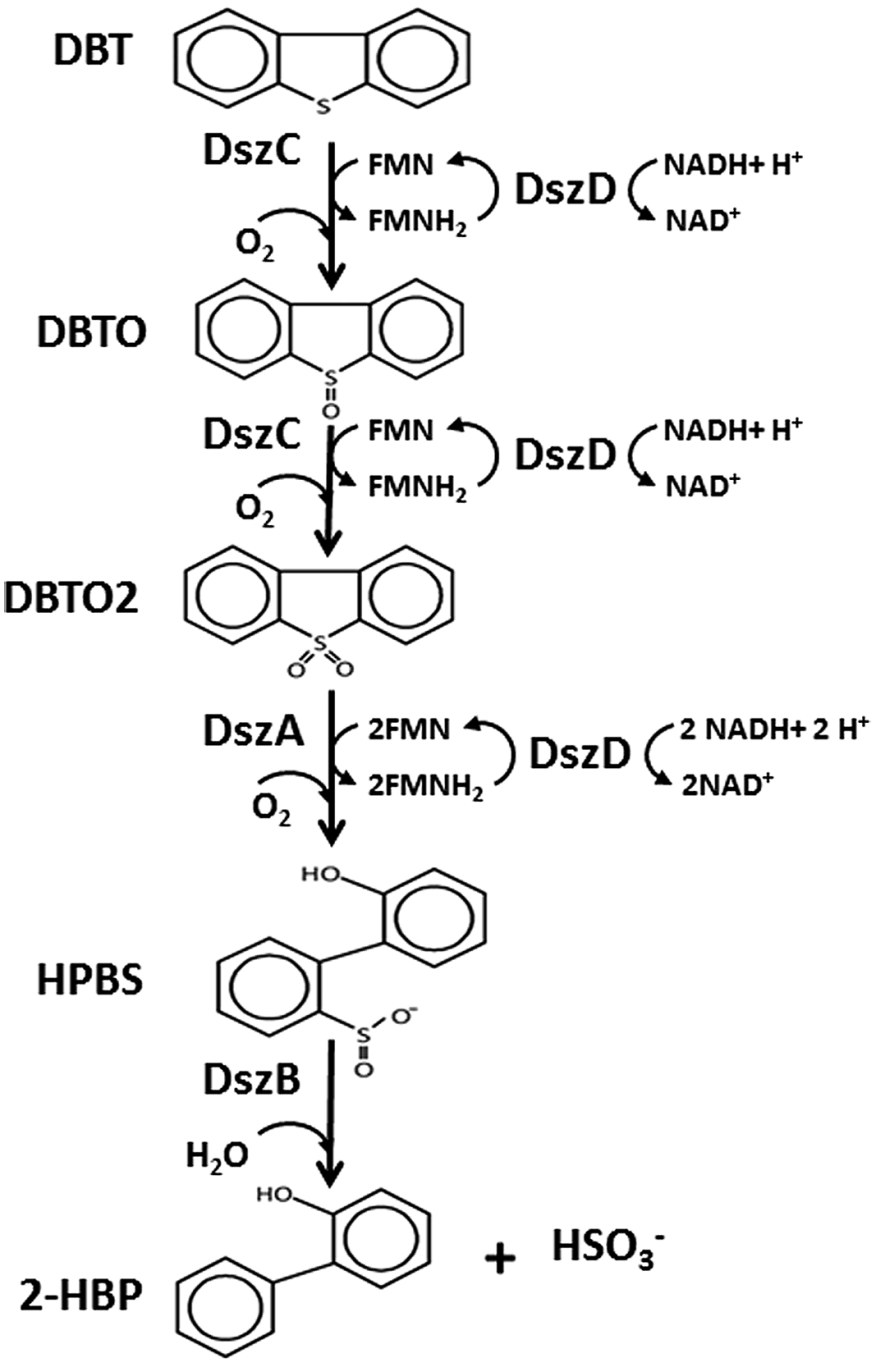
**The 4S pathway involved in desulfurization of DBT into 2-HBP and sulfite mediated by the activity of two monooxygenases (DszC, DszA), a desulfinase (DszB) and an oxidoreductase (DszD).** DszC oxidizes DBT in two sequential steps forming DBTO and DBTO_2_, DszA catalyzes the oxidative C-S bond cleavage in DBTO_2_ forming HBPS, DszB catalyzes the conversion of HPBS into 2-HBP and sulfite (HSO_3_^-^). DszD delivers the reducing equivalent (FMNH_2_) required for the function of DszC and DszA.

The 4S pathway operating in the desulfurization of DBT, which is an oxidative pathway, involves cleavage of the C-S bond in the thiophene ring and the formation of intermediates, which are DBT sulfoxide (DBTO), DBT sulfone (DBTO_2_), 2-(2^′^-hydroxyphenyl) benzene sulfinate (HPBS), 2-HBP and sulfite. The formed 2-HBP is the C-skeleton remaining after removal of S from DBT, and it is not metabolized and accumulates in culture supernatant as dead end product. The 4S pathway has been discovered in some bacterial genera and it is merely a metabolic pathway to deliver bioavailable S for microbial growth from thiophenic compounds. All the bacterial isolates under this study grew well and utilized DBT as the sole S-source. GC analysis of cell extracts as well as culture supernatant revealed the presence of DBT sulfone (DBTO_2_) and 2-HBP, which are real intermediates of the 4S pathway involved in DBT desulfurization. The concentration of 2-HBP increased gradually during the time course of DBT consumption while DBTO_2_ was transiently detected. Molecular detection of the plasmid-borne *dsz* operon, which codes for the enzymes expressing the DBT desulfurization activity, revealed the presence of the *dszA*, *dszB*, and *dszC* genes in SA11 and SA31 in agreement with the reported size and organizational order in other DBT desulfurizing bacteria. Therefore, the desulfurization system in the newly isolated SA11 and SA31 is organized as one operon with three genes (*dszA, dszB, dszC*) transcribed in the same direction and under the control of a single promoter as has been reported in all bacteria exhibiting DBT desulfurization phenotype (Gray et al., 1996; Oldfield et al., 1997). The *dsz* genes were also detected in SA21; however it seems that the set of primers employed are not specific enough for the detection *dsz* genes in this isolate. These data, together with those from GC analysis, are consistent with the view that the bacterial isolates enriched in this study follow the well-known 4S pathway for desulfurization of DBT (**Figure 6**). The expressed DBT desulfurization rate of the isolated SA11 strain is comparable with those reported rates for other wild type bacteria (**Table 2**) and with some others reported previously (Kilbane, 2006). However, it should be emphasized that in some reports the desulfurization rate was calculated based on the amount of consumed substrate (DBT) while in some other reports it was calculated based on the amount of accumulated end product (2-HBP). Moreover, other discrepancies in comparing the desulfurization rates (BDS rates) arise from the utilization of different nutrient media with varying C-sources as well as from the utilization of single- or two-phase BDS protocols which affect the mass transfer and bioavailability of the substrate. Likewise, it has been documented that BDS active bacteria harboring identical *dsz* genes exhibit different desulfurization rates because of the influence of some other host specific factors (Kilbane, 2006; Aggarwal et al., 2012, 2013). Accordingly, a fair comparison of desulfurization rates among different bacterial groups requires the establishment of a standard protocol**.** 2-HBP production lagged behind DBT consumption and cell growth and the amount of the detected 2-HBP was not stoichiometric to the amount of DBT consumed. Only 60–75% of the consumed DBT was recovered in culture supernatant as 2-HBP. Similar observation has been reported and it was suggested that either the retention of 2-HBP by the bacterial cells or/and the accumulation of other intermediates of the 4S pathway would account for this difference (Abin-Fuentes et al., 2013).

**Table 2:** Biodesulfurization specific activity (μmol/g DCW/h) by growing (G) or resting (R) cells of some reported wild bacterial cultures (for other wild and genetically improved BDS bacteria see Kilbane, 2006).

Bacteria	C-source for cell growth/energy	Status of cells (G/R)	BDS specific activity	Reference
*Rhodococcus* sp. strain SA 11	Glycerol	G	11	This study
*Rhodococcus erythropolis XP*	Glycerol/Glucose	G	4	Yu et al. (2006)
*R. erythropolis* strain SHT87	Glycerol	R	21.6	Davoodi-Dehaghani et al. (2010)
*R. erythropolis* LSSE8-1	Glycerol	G	14.1	Li et al. (2009)
*R. erythropolis* DRA	Glucose	R	26	Li et al. (2008)
*R. erythropolis* R1	Glycerol	G	45	Zahra et al. (2014)
*R. globerulus* DAQ3	Fructose	G and R	17–18	Yang et al. (2007)
*Gordonia alkanivorans* strain 1B	Glucose	G	1.6	Alves et al. (2007)
*G. alkanivorans* RIPI90A	Na-benzoate	R	5.1	Mohebali and Ball (2008)
*Microbacterium* sp. Strain ZD-M2	Glycerol	G	11	Chen et al. (2008a)
*Mycobacterium* sp. ZD-19	Glycerol	G	3.7	Chen et al. (2008b)
*Sphingomonas subarctica* T7b	Glucose	R	1.6	Gunam et al. (2013)

The observed rapid drop in bacterial growth when the concentration of the accumulated 2-HBP is around 0.3 mM is consistent with the observed inhibition when 2-HBP was added to growing cultures and suggests feedback inhibition as has been described for other DBT-desulfurizing bacteria (Omori et al., 1992; Chen et al., 2008a). SA11 isolate was the least inhibited biocatalyst and retained around 70% of the growth at 0.3 mM 2-HBP. Recently, our detailed investigation on the mechanism of inhibition of microbial desulfurization, using *R. erythropolis* IGTS8, has confirmed that 2-HBP is the major inhibitor for the enzymes of the BDS process and that the concentration of 2-HBP that led to a 50% reduction in the enzymes’ activity (IC_50_S, 60, 110, and 50 μM, for DszA, DszB, and DszC, respectively) is much less than the cytoplasmic 2-HBP concentration (260–1,100 μM, Abin-Fuentes et al., 2013). The delay in formation of 2-HBP and its lower rate of formation compared to the rate of DBT consumption could be considered as a regulatory tool to avoid fast formation of high concentration of this toxic phenolic compound, which can damage cell membranes. The conversion of 2-HBP into at least two different hydroxynitrobiphenyls by the DBT-desulfurizing *Corynebacterium* strain SY1 possibly is a detoxification mechanism of 2-HBP (Omori et al., 1992), which supports this hypothesis. Apparently, the cells can withstand up to 0.3–0.4 mM of 2-HBP in culture supernatant. This will prolong the viability and avoid harming the cells after an energetically expensive metabolic process (4 moles of NADH are consumed/mole of DBT), which has no energy gain from the accumulated C-skeleton of the desulfurized substrate (Aggarwal et al., 2013).

### VIABILITY AND BDS ACTIVITY OF BIOCATALYST IN CRUDE OILS

Testing the viability of SA11 in environment containing different concentrations of crude oil revealed two distinct phases in terms of the number of recovered cells (expressed as CFU obtained after 48 h incubation), an early phase with decrease in the number of recovered cells followed with a phase with increase in the number of recovered cells. It seems that the number of recovered cells (CFU), in both phases, is inversely proportional to the oil concentration in the assay. The difficulty in recovering cells from the tight emulsion formed at high concentration of oil and/or the death of cells due to the presence of toxic chemical(s) in crude oil are plausible factors influencing the number of recovered cells. In general, apparently the starting inoculum passed through a phase of adaptation to the oily environment where the labile cells die (phase with decrease in recovered cells) followed by a phase where tolerant cells or clones in the inoculum start to flourish and dominate (phase with increase in recovered cells). Overall, the substantial increase in the number of recovered cells after 10 days compared with the starting inoculum indicated the endurance and adaptability of the bacterial cells to live and propagate in environment containing high oil concentrations (50% oil). All cells recovered from crude oil assays retained the BDS activity. The competence of SA11 isolate to survive in heavy crude oil and to retain the BDS activity against DBT point out to the possibility of developing this biocatalyst to design BDS process. It is likely that the finally purified SA11 isolate may have acquired random gene mutations during the time course of enrichment under the stress of DBT which resulted in better desulfurization rate and improved tolerance against 2-HBP.

### BDS OF HEAVY CRUDE OIL

The DBT desulfurizing SA11 biocatalyst exhibited significant capability to desulfurize heavy crude oil. The reduction in total S present in the hexane-soluble fraction (de-asphaltened oil fraction), which contains nearly 70% of the total organic S encountered in crude oil, was higher than in the whole crude oil. This suggests that the bioavailable S needed for growth of SA11 cells in crude oil environment most likely was delivered from desulfurization of DBT and BT compounds, which constitute the majority of S compounds present in oil (Monticillo and Finnerty, 1985). S-speciation analysis of SA11-treated hexane-soluble fraction confirmed the broad desulfurization spectrum, which extended to include DBT and BT as well as wide range of their alkylated derivatives. Such a feature has been the reason to conduct extensive studies to develop a bioprocess for removal of HDS-refractory S species from different refinery streams (see introduction). BDS of crude oils and oil fractions has been also reported by various bacterial species (Van Hamme et al., 2003; Gupta et al., 2005; Soleimani et al., 2007; Mohebali and Ball, 2008). Due to the wide variations in the experimental setup, which involves many variables such as the nature and concentration of oil feedstock in the process; the nature of S compounds in crude oils; the type of biocatalyst; reactor design and bioprocessing scheme; breaking of emulsion and recovery of desulfurized crude; and total S determination; the obtained desulfurization efficiency could not be fairly compared to develop a universal bioprocess for BDS of various crude oils. The longevity and the desulfurization capability of SA11 are crucial traits needed for a robust biocatalyst involved in a pre-refinery bio-process to upgrade crude oils. The measured catalytic desulfurization rate of SA11 is too low and needs to be amplified by at least by two orders of magnitude to develop a commercially feasible bioprocess in oil industry.

### PHYLOGENETIC AFFILIATION OF BACTERIAL BIOCATALYSTS

Alignment with sequences obtained from GenBank showed 99% sequence identity of strain SA11 and SA31 to *Rhodococcus* sp. Strain SA11 was clustered at the clade containing several *Rhodococcus* spp. in consistence with alignment analysis. Strain SA31 was also identified as a member of *Rhodococcus* spp. However, phylogenetically, it was clustered with high bootstrap support far from SA11 indicating a distinct variation between both strains. Although strain SA21 was clustered at the same group with SA31, it has been separated with relatively long phylogenetic branch from it indicating that both strains are less frequently related (**Figure 5**). This finding was supported by the high sequence identity of strain SA21 to *Stenotrophomonas* sp. rather than *Rhodococcus* sp. Based on such phylogenetic analysis, both strains, SA21 and SA31 could be interpreted as new strains belonging to *Rhodococcus* sp. and *Stenotrophomonas* sp. respectively.

The obtained results in this study describe the characterization of newly isolated bacterial strains, which are capable of desulfurizing thiophenic compounds encountered in crude oils. Data were presented confirming the desulfurization of DBT via the well-known 4S pathway. One of the bacterial isolates, which was affiliated as *Rhodococcus* sp. strain SA11, was capable of surviving in heavy crude oil and exhibited wide desulfurization spectrum against thiophenic compounds including DBTs and BTs. Moreover, the SA11 strain showed reasonable tolerance against the inhibitory effect of 2-HBP. These remarkable biocatalyst features are important for the development of bioprocesses for upgrading crude oils. Further functional genomic characterization of the BDS activities against DBT, BT, and their homologous alkylated derivatives by SA11 strain as well as the detailed desulfurization spectrum of S-compounds encountered in crude oils are undergoing.

## Conflict of Interest Statement

The authors declare that the research was conducted in the absence of any commercial or financial relationships that could be construed as a potential conflict of interest.

## Footnotes

1 http://www-igbmc.u-strasbg.fr/BioInfo/

2 ftp://ftp.ebi.ac.uk/pub/software/clustalw2

3 http://www.gastechnology.org
